# The facial microbiome and metabolome across different geographic regions

**DOI:** 10.1128/spectrum.03248-23

**Published:** 2023-12-08

**Authors:** Rong Tao, Tingting Li, Yalin Wang, Rong Wang, Ruoyu Li, Pascale Bianchi, Hélène Duplan, Ying Zhang, Hang Li, Ruojun Wang

**Affiliations:** 1 Department of Dermatology, Beijing Tongren Hospital, Capital Medical University, Beijing, China; 2 Department of Dermatology of Peking University First Hospital, National Clinical Research Center for Skin and Immune Diseases, Beijing Key Laboratory of Molecular Diagnosis on Dermatoses, NMPA Key Laboratory for Quality Control and Evaluation of Cosmetics, Beijing, China; 3 Affiliated Suzhou Hospital of Nanjing Medical University, Suzhou, China; 4 Hexi University Affiliated Zhangye People’s Hospital, Zhangye, Gansu, China; 5 Department of Research & Development, Pierre Fabre Dermo-Cosmetic & Personal Care, Boulogne-billancourt, France; 6 Medical Department, Pierre Fabre Dermo-Cosmetic, Shanghai, China; University of California San Diego, La Jolla, California, USA

**Keywords:** microbiome, metabolome, clinical study

## Abstract

Facial skin is more exposed to the outside environment, and it is usually affected by environmental factors. Characterization of the skin microbiome and metabolome of individuals from different geographic regions will help to understand the factors behind the prevalence of skin disorders and support skincare product development. In the current study, volunteers from northern, southern, and northwestern China were recruited. Facial skin samples were collected for amplicon sequencing and metabolomic liquid chromatography‒mass spectrometry (LC–MS/MS) analyses. We demonstrated significant alterations in the abundances of multiple microorganisms and skin lipid compositions between individuals. People living in the northwest, which has a dry climate and high altitude, showed lower level of total lipids, and lower abundance of *Malassezia*, but higher levels of ceramides and fatty acids. *Malassezia* had a positive correlation with eicosanoids and a negative correlation with ceramides. This work highlights the effects of geographic factors on the skin microbial structures and skin lipids.

**IMPORTANCE:**

Characterization of the skin microbiome and metabolome across geography will help uncover the climate factors behind the prevalence of skin disorders and provide suggestions for skincare products for people living in different geographic regions.

## INTRODUCTION

Following a landmark study on the topographical and temporal diversity of the human skin microbiome, a growing number of studies have focused on this area (1). Several infectious, inflammatory, and neoplastic dermatoses are associated with shifts in the skin microbial composition (2). Until now, single-center studies have profiled the human skin microbiome under healthy and diseased states, whereas there is a lack of studies on the effects of environmental factors on the healthy microbiome (3).

Local host factors, systemic host factors, lifestyle factors, and environmental factors together contribute to the colonization of microorganisms on the skin (4). Human facial surface lipids, consisting of ceramides, fatty acids, triglycerides, and cholesterol, are crucial local host factors for maintaining a healthy skin microbiome and skin barrier, and their disequilibrium is correlated with barrier dysfunction and inflammation (5 – 7). Interestingly, recent research revealed that the microorganisms and lipids on the skin have reciprocal effects. Host-derived lipids, such as fatty acids and sphingoid bases, have antimicrobial activity, whereas dysregulated microbes and microbial metabolites may lead to barrier damage (8, 9). Facial skin is frequently affected by ambient factors, such as climate, temperature, humidity, ultraviolet exposure, and pollution, since it is more exposed to the external environment than skin on other parts of the body (5). Climate factors are considered to play crucial roles in the occurrence of various skin disorders, leading to disparities in their prevalence in different regions (10). Nevertheless, how environmental factors influence the skin microbiome and metabolites has been poorly studied.

Herein, the aim of this was to investigate and compare the skin microbiome and metabolome of healthy facial skin among individuals living in different geographic regions in China. This study will help to better comprehend the effects of environmental exposures on skin microbial disturbance in disease states.

## MATERIALS AND METHODS

### Subject recruitment and sample collection

We enrolled 25 volunteers from Beijing in North China, 23 volunteers from Zhangye, Gansu Province in Northwest China, and 23 volunteers from Suzhou, Jiangsu Province in South China between August 2022 and October 2022. To reduce the impact of potential confounding factors, all participants were of Han nationality, indoor workers and living in urban areas. The average ages and sex ratios were comparable among individuals from the northern (mean ± SD: 35.55 ± 12.65; male:female 1:1), northwestern (mean ± SD: 29.22 ± 6.12; male:female 9:14), and southern regions (mean ± SD: 33.62 ± 8.65; male:female 9:12).

Inclusion criteria included: aged between 18 and 60 years, with no active facial dermatoses or severe systemic diseases, such as autoimmune diseases, malignancies, diabetes, etc. To avoid potential confounding factors, only people of Han nationality who were indoor workers and lived in urban areas were recruited. The exclusion criteria included the use of antibiotics or antifungal agents 12 weeks prior to the study; the use of topical therapeutic products on the scalp within 4 weeks prior to the study; and the long-term use of systemic steroids or immunosuppressants.

Volunteers were advised not to wash their face for 24 hours prior to the sampling procedure. Skin swabs were obtained from the cheeks using a sterile cotton swab. The swab was premoistened in phosphate buffer saline (PBS) solution and rubbed onto the skin surface for 10 times. At the end of the procedure, the head of each swab was cut from the handle and placed into two tubes containing 1.5 mL of PBS. Samples for amplicon sequencing were stored at −20°C before DNA extraction and samples for metabolome analysis were stored at −80°C. Samples were transported to Peking University First Hospital by cold chain facility using dry ice before further processing.

### DNA extraction

All specimens were incubated in preparation with lysis buffer and lysozyme (20 mg/mL) for 30 minutes at 37°C, then mechanically disrupted using 5 mm stainless steel beads (Qiagen, Hilden, Germany) in a Tissuelyser (Qiagen) for 2 minutes, 30 HZ. DNA was extracted from the skin swabs using the DNeasy Blood and Tissue kit (Qiagen, Hilden, Germany) following the manufacturer’s instructions. The extracted DNA quality and concentration were evaluated using a spectrophotometer (Nanodrop 2000, Thermo Fisher Scientific, Waltham, MA, USA), and the DNA was stored at −80°C prior to polymerase chain reaction (PCR) analysis.

### Amplicon sequencing and data processing

For bacterial microbiome analysis, in order to have higher taxonomic resolution of the reads at the species level, we amplified the nearly full-length of 16S rRNA genes using specific primers (forward: 27F 5′-AGAGTTTGATCCTGGCTCAG-3′; reverse: 1492R 5′-TACGGYTACCTTGTTACGACTT-3′) with the barcode. All PCRs were carried out with TransStart FastPfu DNA Polymerase (TransGen Biotech, Beijing, China). The PCR products were mixed with an equal volume of operate electrophoresis on 2% agarose gel for detection, then purified with QIAquick@Gel Extraction Kit (Qiagen). Sequencing libraries were generated using the SMRTbellTM Template Prep Kit (PacBio), and the library quality was assessed with the Qubit@ 2.0 Fluorometer (Thermo Scientific, Waltham, USA) and FEMTO Pulse system. The library was sequenced on the PacBio Sequel platform by Novogene Bioinformatics Technology Co., Ltd. (Beijing, China).

Raw sequences were processed through the PacBio SMART portal. Sequences were filtered with a threshold of minimum predicted accuracy of 90%. Amplicon size trimming was performed to remove sequences outside the expected amplicon size (min.Length 1,340 bp, max.Length 1,640 bp), and the reads were assigned to samples based on their unique barcode. Chimera sequences were detected using the UCHIME algorithm and removed. Sequences with ≥97% similarity were assigned to the same OTUs, and the SSUrRNA Database of Silva Database was used for species annotation based on the Mothur algorithm to annotate taxonomic information.

For the fungal microbiome, DNA was amplified with primers specific for the fungal internal transcribed spacer (ITS)1 rDNA gene (forward ITS5-1737F: 5′-GGAAGTAAAAGTCGTAACAAGG-3′; reverse ITS2-2043R: 5′-GCTGCGTTCTTCATCGATGC-3′). PCR products were separated by electrophoresis in agarose gels (2%, wt/vol) and purified with a Universal DNA Purification Kit (TianGen, China). The amplicon library was prepared using a TruSeq DNA PCR-Free Sample Preparation Kit (Illumina Inc., San Diego, CA, USA) and quantified by Qubit and quantitative PCR analyses. Amplicon sequencing was performed on an Illumina HiSeq 2500 platform.

Paired-end reads were merged using FLASH (V1.2.11, http://ccb.jhu.edu/software/FLASH/), and quality filtered by the fastp (Version 0.23.1) software (11). The tags were compared with the Unite Database (https://unite.ut.ee/) for chimera detection and removal. The effective tags were denoised using DADA2 and annotated with the Unite database in the QIIME2 software.

### Microbiome data analysis

All the data were analyzed in the R software (Version 4.3.0). Differences in microbial communities were assessed using the Wilcoxon rank sum test. Analysis of alpha diversity indexes and nonmetric multidimensional scaling (NMDS) was performed using the vegan package in R. Spearman correlation analyses were carried out using the pheatmap package in R. A false discovery rate correction was applied to adjust the level of significance, and differences were considered significant at *P* < 0.05.

### Metabolomic sequencing and analysis

The liquid samples (100 µL) were added to Eppendorf safe-lock tubes and resuspended in 400 µL of solution [acetonitrile:methanol = 1:1 (v:v)] containing an internal standard to extract metabolites. Then, the samples were mixed, sonicated, and placed at −20°C to precipitate the proteins. The supernatant was blow-dried under nitrogen, resolubilized with 100 uL 50% acetonitrile, and extracted by ultrasonication. Finally, the solution was transferred into the liquid chromatography‒mass spectrometry (LC‒MS/MS) system for analysis.

The LC‒MS/MS analyses were conducted on a Thermo UHPLC-Q Exactive HF-X system equipped with an ACQUITY HSS T3 column at Majorbio Bio-Pharm Technology Co. Ltd. (Shanghai, China). A pooled quality control sample was prepared by mixing equal volumes of all samples. The mass spectrometric data were collected using a Thermo UHPLC-Q Exactive HF-X Mass Spectrometer equipped with an electrospray ionization source operating in positive mode and negative mode (12).

The raw data files generated by LC‒MS/MS were processed using Progenesis QI (Waters Corporation, Milford, USA). The metabolites were identified by searching the HMDB (http://www.hmdb.ca/), Metlin (https://metlin.scripps.edu/), and Majorbio Database. A free online platform of majorbio cloud platform (cloud.majorbio.com) was used for analysis. The R package mdatools was used to perform least partial squares discriminant analysis (PLS-DA). The total levels of lipids, fatty acids, ceramide, and eicosanoids were calculated by summing up the levels of the following classes: lipids—“lipids and lipid-like molecules” (HMDB superclass); fatty acids—“fatty acids and conjugates” and “fatty acid esters” (HMDB class); ceramide—“ceramides” (HMDB subclass); eicosanoids—“eicosanoids” (HMDB subclass). The metabolites with variable importance in the projection (VIP) >1 and *P* value <0.05 were determined to be significantly different metabolites. Spearman correlations were performed for correlation analyses between microorganisms and metabolites. All the data were displayed in R software.

## RESULTS

### Characterization of climates

**Fig 1:**
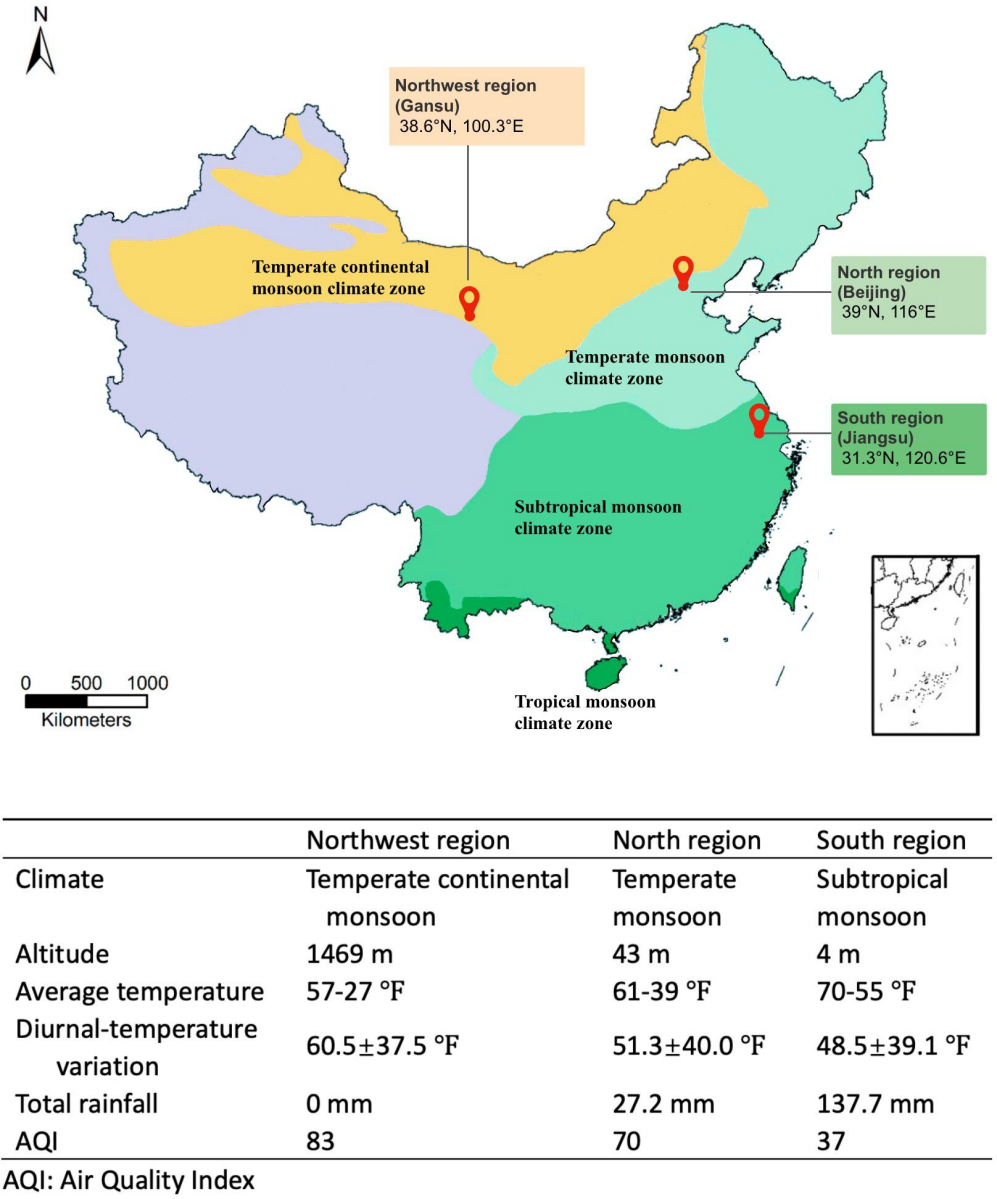
Locations and climates of different geographic regions [map adapted from *Tropical Conservation Science* (15)].

The three geographic regions are representative of three major climates in China (13, 14). Gansu, in the northwest plateau, has a temperate continental monsoon climate with hot and dry summers, cold and dry winters, low annual precipitation, and high ultraviolet radiation. Suzhou, which is in the south, has a subtropical monsoon climate with hot and humid summers, warm and wet winters, high annual precipitation, and lower ultraviolet radiation. Beijing, in northern China, has a temperate monsoon climate, which is characterized by hot and humid summers, cold and dry winters, and relatively moderate annual precipitation and ultraviolet radiation (Fig. 1). During the period of sampling (between August and October 2022), the diurnal variation in temperature is highest in the northwest, followed by the north, while the south located in a low-lying humid area showed low diurnal variation.

### Bacterial community profiles between regions

**Fig 2:**
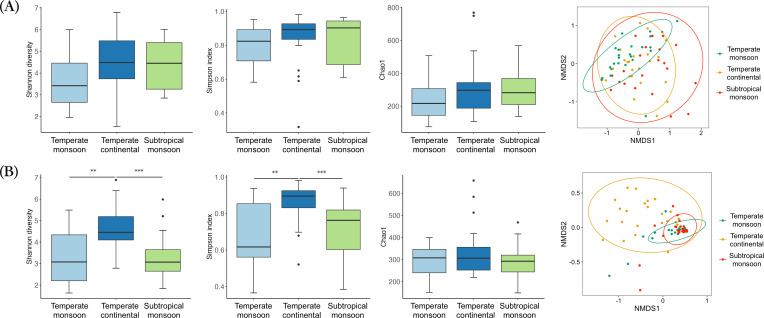
The Shannon diversity, Simpson index, Chao1, and NMDS plot of bacterial (**A**) and fungal (**B**) communities in different geographic regions.

The Shannon diversity, Simpson, and Chao1 indexes of the bacterial community were not different between different geographic regions. NMDS analysis of the bacterial community did not reveal distinct clustering among groups (Fig. 2).

**Fig 3:**
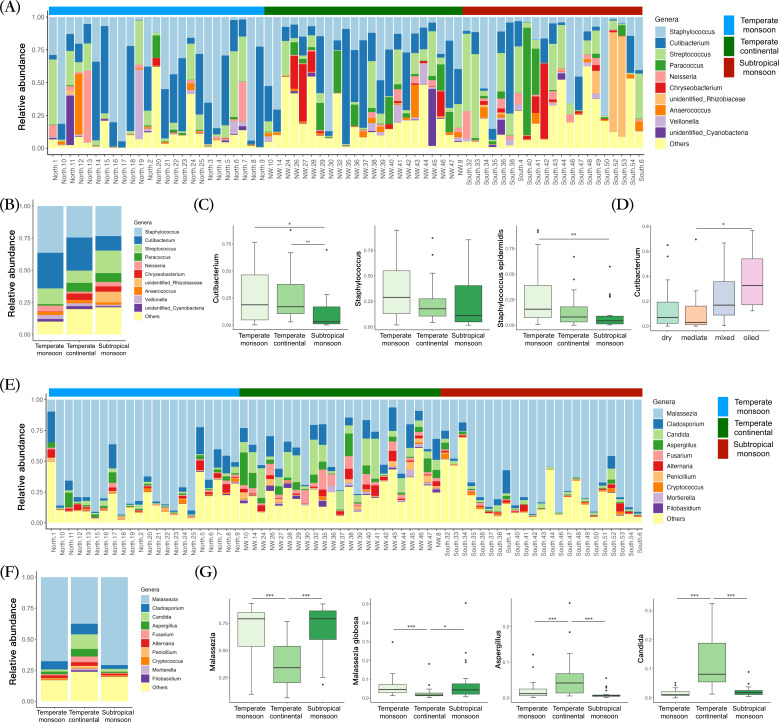
(**A**) The bacterial community structures of samples from different geographic regions. (**B**) The bacterial community structures of different regions. (**C**) The relative abundances of *Cutibacterium*, *Staphylococcus,* and *S. epidermidis* in different regions were shown. (**D**) The relative abundance of *Cutibacterium* in subjects with different skin types. (**E**) The fungal community structures of samples from different geographic regions. (**F**) The fungal community structures of different regions. (**G**) The relative abundances of *Malassezia*, *Aspergillus*, *Candida,* and *Malassezia globosa* in different regions were shown.

Analysis of bacterial taxa revealed significant alterations in face bacterial community composition among different living environments and climates (Table S1). Firmicutes, Actinobacteria, and Proteobacteria were the predominant bacterial phyla on the facial skin in all geographic regions. At the genus level, the relative abundances of *Staphylococcus* and *Cutibacterium* were significantly higher in samples from the north. *Cutibacterium*, a lipophilic bacterium, showed higher relative abundance in subjects with the oiled skin type than in those with other skin types. Species-level analysis revealed that *Staphylococcus epidermidis* was more abundant in samples from the north than in those from other regions (Fig. 3A). Further correlation analysis showed that age was positively correlated with *Chryseobacterium* and negatively associated with *Staphylococcus* and *Cutibacterium*.

### Changes in fungal community structures in the plateau region

Significantly higher fungal Shannon diversity and Simpson index values were observed in the northwestern region than in other regions, indicating variation in the fungal microbiome in the plateau. NMDS analysis of the fungal community showed that samples from the northwest were more discrete than those from the north and south.

Basidiomycota and Ascomycota were the dominant fungal phyla on the facial skin. At the genus level, *Malassezia* was found to dominate the fungal community in all samples, and its relative abundance was significantly higher in samples from the north and south than in those from the northwest. The relative abundances of *Cladosporium*, *Candida*, *Aspergillus*, *Fusarium*, *Alternaria*, *Penicillium*, *Mortierella,* and *Filobasidium* were significantly increased in samples from the northwest compared with those from other regions. At the species level, we observed a significant decrease in the relative abundance of *M. globosa* in samples from the northwest (Fig. 3B; Table S2). Correlation analysis showed that female sex was correlated with a higher proportion of *Malassezia* on the skin, whereas skin type was not correlated with *Malassezia*.

### Altered skin lipid composition between geographic regions

**Fig 4:**
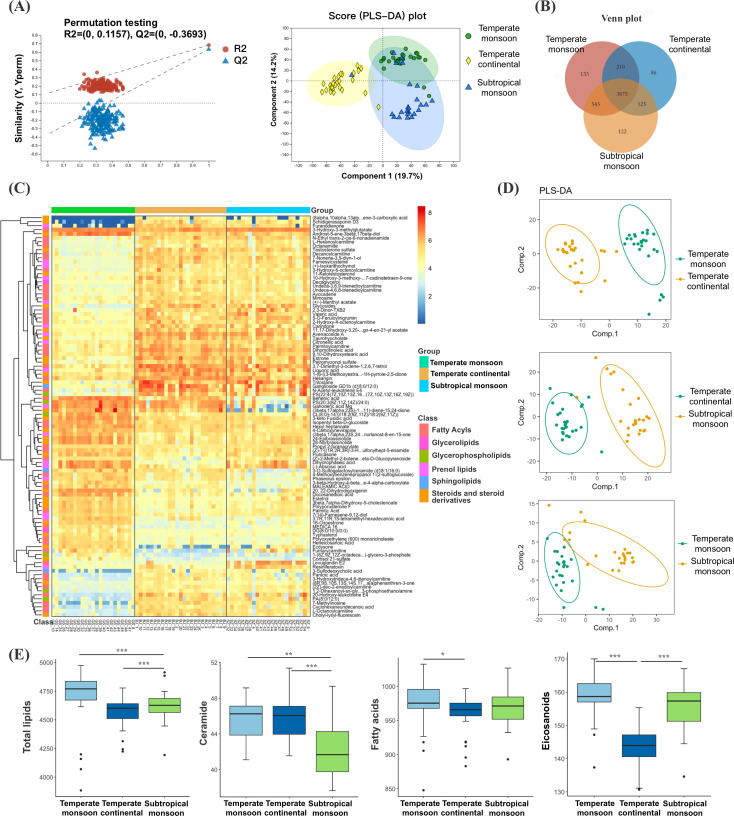
(**A**) PLS-DA results of metabolites between geographic regions. (**B**) Venn plot of all metabolites. (**C**) Heatmap of differentially expressed metabolites. (**D**) PLS-DA results of lipids and lipid-like molecules. (**E**) Levels of total lipids, ceramides, fatty acids, and eicosanoids between geographic regions.

Metabolome analysis of skin samples using a nontargeted LC‒MS-based metabolomics approach was performed. PLS-DA, which is a supervised multivariate clustering or classification method, was used to cluster the samples, and the model results revealed a separation between samples from the northwest and those from other regions, whereas samples from the north partially overlapped with those from the south (Fig. 4A). A total of 4,121 common metabolites were found in all groups, whereas each region presented with numerous unique metabolites (Fig. 4B; Table S3).

To investigate the effect of climate and environmental factors on skin barrier composition, we focused on skin lipids, especially fatty acids and ceramides. A heatmap was generated as a graphical representation of the differentially expressed lipids (Fig. 4C). The model results of PLS-DA conducted based on lipids and lipid-like molecules showed clearly distinct samples from each geographic region (Fig. 4D).

Further analysis showed that the ceramide level was lower in skin samples from the southern region than in those from the northern and northwestern regions. The total level of fatty acids was lower in samples from the northwest than in those from the north. Several long-chain fatty acids, such as palmitic acid, docosanedioic acid, oleic acid, and behenic acid, were significantly enriched in samples from the northwest compared to other regions. However, the levels of total eicosanoid and its several family members, prostaglandins, and leukotrienes, which are considered mediators of allergic diseases, were significantly decreased in samples from the northwest (Fig. 4E).

### Correlation between microorganisms and lipids

**Fig 5:**
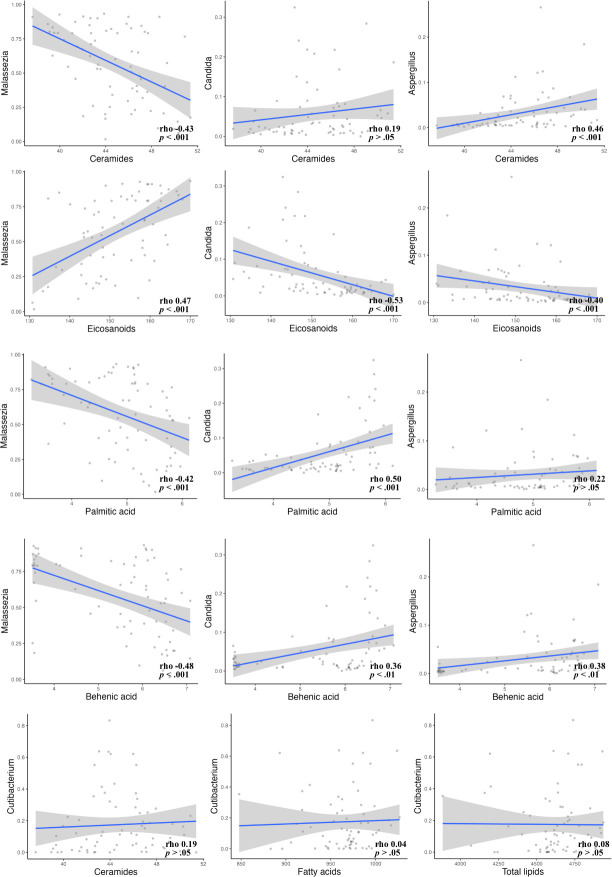
Correlation analysis between skin microorganisms and skin lipids, fatty acids, ceramides, and eicosanoids.

We analyzed the correlation between microorganisms and skin lipids, including fatty acids, ceramides, and eicosanoids. *Malassezia*, a lipophilic yeast, was strongly positively correlated with eicosanoids (Spearman correlation, rho 0.47, *P* < 0.001) and several medium- to long-chain fatty acids, such as behenic acid and palmitic acid; however, it was negatively correlated with ceramides (rho 0.43, *P* < 0.001). In contrast, other skin residential microorganisms, such as *Candida*, *Aspergillus*, *Alternaria*, *Cladosporium,* and *Lactobacillus*, showed opposite correlation trends, including a positive correlation with ceramides and a negative correlation with eicosanoids. *Cutibacterium*, another well-known lipophilic bacterium, was not correlated with total lipids, fatty acid derivatives, or ceramides (Fig. 5).

## DISCUSSION

In this study, we revealed significant alterations in both bacterial and fungal community structures and skin metabolites, especially the composition of lipid and lipid-like molecules, between individuals living in different geographic regions and climates. Facial skin samples from the northwest, which is in the plateau with a temperate continental monsoon climate, showed higher ceramides, lower eicosanoids, and lower levels of total lipids, along with a decreasing trend of *Malassezia*, in comparison to those from the flat regions (north and south).

Microbiome analysis revealed that the southern regions, with a warm and wet climate, are associated with higher bacterial diversity and higher levels of *Malassezia*. Studies have shown that skin bacteria prefer warmer temperatures, with an optimal growth temperature of 33.2–35.0°C, close to the skin surface temperature (16). Sebum secretion climbs by 10% for each degree at which the temperature rises. As a result, lipid-dependent organisms such as *Malassezia* and *Cubibacterium* thrive in warm environments (17). However, the inconsistent changing trend of *Malassezia* and *Cutibacterium* between different regions in this study might be attributed to other factors, such as rainfall, humidity, air pollution, UV exposure, and diurnal-temperature variations. The overall bacteria survive longer and show higher diversity in an environment with higher humidity (18). UV exposure has also been proven to shape the skin microbiome, including alterations in the abundance of specific bacteria, although the underlying mechanisms are still unclear (17, 19). In addition, high diurnal variations in temperature may also shape the skin microbiome since some microbes are more sensitive to changes in environmental conditions.

Acne and allergic dermatitis are the most frequent disorders that affect facial skin. The northern region had the highest incidence rate of acne (67.67%), followed by the southern region (55.51%), and the northwestern region (46.43%), which was consistent with the relative abundance of *Cubibacterium* in our results (20). Fungal spores are known to be associated with allergy diseases. The prevalence of allergic diseases was much lower in the northwest than in the south, probably due to increased humidity that triggers spore production and dissemination in the south (21). The differences in the microbiome and the prevalence of related diseases in different climate regions suggest that climate and environmental factors play an important role in facilitating health or disease by balancing or disturbing the microbiome.

Previous studies have identified that skin surface lipids, consisting of ceramides, fatty acids, triglycerides, and cholesterol, are of great importance for maintaining a healthy skin condition (22). Here, we add new evidence that the cutaneous lipid composition varies by geographical and climatic conditions. Ceramides are essential constituents in the formation of an epidermal permeability barrier, whereas eicosanoids are mediators of allergy and inflammation diseases (23, 24). People living in the northwest seem to have a better skin barrier than those living in other regions, with increased ceramides and decreased eicosanoids. This finding was consistent with the lower prevalence of allergic diseases in the northwest (21, 25, 26). The effects of climate on skin lipids are unclear. We suppose that the high UV exposure in plateau regions improves the skin barrier by promoting an increase in the synthesis of ceramides and fatty acids, and dry weather may maintain skin lipids by reducing the sweating and wash frequency of the face (27). In addition, shifts in skin microbiome compositions may also partially explain alterations in skin surface lipid compositions. Several *Malassezia* species produce a great abundance of eicosanoids, and *S. epidermidis* secretes a sphingomyelinase that aids host production of ceramides (28 – 30).

This study is mainly limited by its observational nature. Further longitudinal studies will better demonstrate the dynamic microbiome signatures of facial skin. Another limitation is a lack of skin physiological parameters, which will help to evaluate the skin barrier in different areas and correlate them with the microbiome and metabolome. In addition, it is difficult to analyze the effect of one specific geographical factor on the skin microenvironment. We have made efforts to reduce intragroup variance by enrolling participants in the same season, and having them all belong to the Han nationality, have similar living habits, and inhabit urban areas of China.

In conclusion, this study illustrates that geographic factors affect both the skin microbial composition and metabolites. Further research is needed to explore the environmental factors that contribute to the skin microbiome and metabolome and their potential impact on skin health.

## Data Availability

Sequence data from this study have been submitted to GenBank under accession number PRJNA997437.

## SUPPLEMENTAL MATERIAL

Table S1 spectrum.03248-23-s0001.xlsx Relative abundances of bacteria at genus and species levels.

Table S2 spectrum.03248-23-s0002.xlsx Relative abundances of fungi at genus and species levels.

Table S3 spectrum.03248-23-s0003.csv Levels of metabolites in all samples.

ASM does not own the copyrights to Supplemental Material that may be linked to, or accessed through, an article. The authors have granted ASM a non-exclusive, world-wide license to publish the Supplemental Material files. Please contact the corresponding author directly for reuse.
